# Editorial: Targeting antigen processing and presentation in autoimmune and autoinflammatory disorders

**DOI:** 10.3389/fimmu.2022.1055152

**Published:** 2022-10-13

**Authors:** Patrizia Leone, Vito Racanelli

**Affiliations:** ^1^ Department of Biomedical Sciences and Human Oncology, School of Medicine, ‘Aldo Moro’ University of Bari, Bari, Italy; ^2^ Department of Interdisciplinary Medicine, School of Medicine, ‘Aldo Moro’ University of Bari, Bari, Italy

**Keywords:** antigen processing and presentation, autoimmunity, HLA class I and class II typing, proteasome, ERAP

With contribution from environmental triggers, the development of autoimmune disease is thought to be due to genetic predisposition and breakdown of self-tolerance mechanisms.

Collection presents recent studies covering defects in antigen processing and presentation mechanisms and genetic mutations of genes coding for proteins involved in this process underlying the onset and progression of autoimmune diseases.

Antigen processing and presentation is a complex biological process resulting in activation of the two functional subsets of T cells, CD8 and CD4 T cells, and in mounting of an adaptive immune response against pathogens. By T cell receptor, T cells only recognize antigen peptides that are displayed on cell surface and are bound to major histocompatibility complex (MHC) class I and class II molecules. These antigen peptides derived from pathogen proteins by two major proteolytic pathways: the ubiquitin-proteasome pathway and the lysosomal pathway (1).

The ubiquitin-proteasome pathway is mainly involved in degradation of cytosolic proteins, such as misfolded and damaged proteins, mutated proteins in cancer cells, and proteins derived from all viruses and intracellular bacteria replicate in the cytosol (endogenous antigens) (2–4). The principal player of this pathway is the proteasome, a highly sophisticated multisubunit protease complex that cleaves ubiquitinated proteins into oligopeptides which are released to the cytosol and transported into the endoplasmic reticulum (ER) to be bound to the MHC class I molecules. The peptide:MHC class I complexes leave the ER and through the Golgi apparatus deliver cytosolic peptides to the cell surface to present them to antigen-specific cytotoxic CD8 T cells (4).

The lysosomal pathway is responsible for breaking down intracellular damaged proteins or organelles taken up by autophagy, and proteins derived from extracellular pathogens engulfed by cells through endocytosis (exogenous antigens). In this pathway, bacteria, bacterial antigens, parasites are taken up into endosomes, acid compartments which can eventually fuse with lysosomes, and are degraded by proteases into peptide fragments that bind directly to MHC class II molecules. The peptide:MHC class II complexes are then shuttled to the cell surface where they present antigens to T helper (Th) CD4 cells (5). Professional antigen presenting cells (APC) can overcome these rules and present exogenous antigens on MHC class I molecules to CD8 T cells, in a process known as cross-presentation. During this process, exogenous antigens are translocated from the phagosome into the cytosol, where they take the way of endogenous antigens. Cross-presentation is required for triggering cytotoxic responses to certain exogenous antigens, such as some viruses do not infect professional APC and cancer cells (6).

Processing of self-proteins and presentation of self-antigens occur in the same manner as foreign antigens and are needed to establish and maintain tolerance. The failure of natural tolerance mechanisms combined with genetic susceptibility and environmental triggers such as infection results in autoimmune disease development.

This Research Topic is dedicated to defects in antigen processing and presentation responsible for ongoing autoinflammatory and autoimmune disorders.

Antigen processing and presentation underlies the development of all autoinflammatory and autoimmune diseases, influencing both tolerance induction in the thymus and self-antigen recognition and immune cell activation in the periphery (**Figure 1**). This is highlighted by the fact that the genetic risk factors for multiple autoimmune diseases are genes involved in antigen processing and presentation. Most important among these are specific human leukocyte antigen (HLA) alleles encoding MHC class I and MHC class II molecules (7–9). For instance, there is a strong association between expression of HLA-DRB1 alleles containing the shared epitope, a conserved amino acid sequence at residues 70 to 74 in the third hypervariable region of the DRβ1 chain, and susceptibility to and severity of rheumatoid arthritis (10, 11). The HLA-DRB1*0301 and -DRB1*0401 alleles confer susceptibility to autoimmune hepatitis type 1 (AIH-1), whereas the possession of the DRB1*07 allele is linked to AIH-2 (12). The HLA-B*27 family of alleles is highly associated with Spondyloarthropathies (SpA) and in particular with the Ankylosing Spondylitis (13).

**Figure 1:**
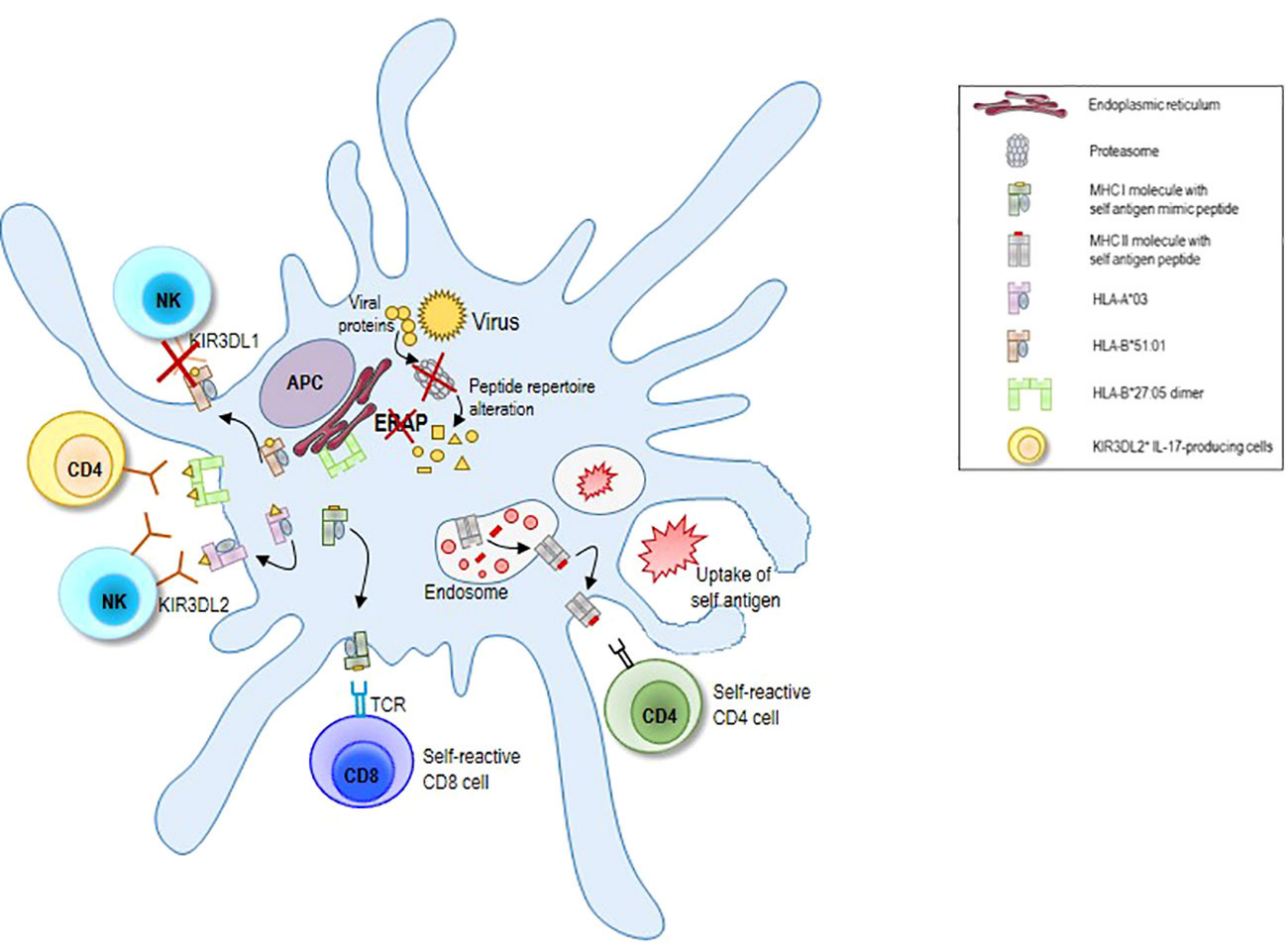
Schematic illustration of the alterations in antigen processing and presentation underlying autoimmune disease development. Mutations of genes coding for proteasome subunits and ERAP molecules can completely change peptide repertoire with consequent alterations in CD8 T cell and NK cell mediated immune responses. Moreover, the abnormal expression of HLA variants can contribute to cause autoimmune responses. Proteasome subunit mutations can generate peptides that mimic self-antigens (molecular mimicry) and that are presented by MHC I molecules to self-reactive CD8 T cells. Loss of ERAP 1 molecules can render HLA-B*51:01 molecules less eligible for binding to KIR3DL1 receptor with consequent NK cell activation and killing of HLA-B*51:01 expressing cells. The interaction between KIR3DL2 receptor and its ligands HLA-A*03 or A*11 results in NK cell inhibition. Beside of peptide repertoire, polymorphisms in ERAP 1 gene may influence HLA-B*27 intrinsic misfolding properties. HLA-B*27:05 free H chain can also generate dimer complexes that can be retained within the ER or transported to the surface where they can interact with the receptor KIR3DL2 on NK cell or on IL-17 producing CD4 cells. This latter mechanism supports survival and differentiation of KIR3DL2^+^CD4^+^ T lymphocytes in patients with ankylosing spondylitis. As result of the breakdown of tolerance mechanisms, APC can also process self-proteins engulfed by endocytosis or autophagy and present self-antigens on MHC II molecules to self-reactive CD4 T cells, leading to autoimmune diseases. APC, antigen presenting cells; MHC, major histocompatibility complex; HLA, human leukocyte antigen; ERAP, endoplasmic reticulum aminopeptidase protein; NK, natural killer cell.

Moreover, there is evidence to suggest that the risk of disease associated with expression of certain MHC proteins is modulated by interactions between many HLA alleles (14). Interactions between classical HLA alleles and non-HLA risk-associated variants have been illustrated for coding variation in the antigen processing ER aminopeptidase protein 1 (ERAP1) gene and the HLA-C*06 in psoriasis (15), HLA-B*51 in Behçet’s disease (16), and HLA-B*27 allele in ankylosing spondylitis (17, 18).

Using a single chain trimeric (SCT) format, composed of a specific peptide fused to the light chain beta-2 microglobulin and MHC class I heavy chain (HC), Lenart et al. have demonstrated that HLA-B27 HC possesses intrinsic misfolding properties. A series of HLA-B27 SCT substitution mutations indicated that the F pocket and antigen binding groove regions influenced the folding and dimerisation of the single chain complex; HLA-B27 SCT molecules showed an ability to form dimers and to vary in their association with the intracellular antigen processing machinery. These findings could explain why, even in the presence of a high affinity HLA-B27 binding peptide, HLA-B27 transgenic rats with a SpA-like phenotype do not show an improvement of gastrointestinal symptoms and that these clinical features may be more due to HLA-B27 misfolding.

Allelic differences of ERAP1 and ERAP2 as well as mutations of genes coding for proteasome subunits can completely change peptide repertoire with consequent alterations in CD8 T cell and NK cell mediated immune responses (D’Amico et al., 19). For instance, loss of ERAP1 makes HLA-B*51:01 molecules less eligible for binding to KIR3DL1 receptor enhancing the NK cell killing of HLA-B*51:01 expressing cells (D’Amico et al.). Alterations of the KIR-HLA repertoire could also impact the response to treatments related to NK cell activity. Individuals carrying HLA-B*51:01-like antigens may be candidate for NK-cell based immunotherapy with ERAP1 pharmacological inhibition. Moreover, Muraro et al. have recently demonstrated the influence of KIR-HLA repertoire on trastuzumab efficiency in patients with HER-2 positive breast cancer. In these patients, the presence of KIR3DL2 and its ligands HLA-A*03 or A*11 is associated with worse outcome.

Alterations of the antigen processing and presentation machinery have also a significant impact on clearance of infections that may predispose to autoimmunity by several mechanisms, including enhanced expression of costimulators in tissues and cross reactions between microbial antigens and self-antigens (molecular mimicry). Recently, Fasano et al. have reviewed molecular mechanisms that underlie pathogenesis of AIH, highlighting the contribute of molecular mimicry and enhanced self-antigen presentation (by standard and alternative mechanisms) in the activation of self-reactive lymphocytes (Th1, Th2, Th17, cytotoxic T lymphocytes and macrophages) and autoimmune responses (Fasano et al., Wang et al.).

Interestingly, Davies et al. have provided new insights into mechanisms that regulate autoimmunity demonstrating that epidermal langerhans cells upregulate antigen presentation genes along with an immunoregulatory module including the genes IDO1, LGALS1, LAMTOR1, IL4I to more efficiently prime regulatory T cells, and maintain immune homeostasis in the skin.

We expect the Research Topic to serve as one-stop overview of the impact of antigen processing and presentation mechanisms and its defects on the autoimmune responses. A better understanding of this issue may open new therapeutic avenues. Antigen specific immunotherapy designed to finely manipulate antigen presentation and restore immunological self-tolerance to self-antigens leaving the rest of the immune system to function successfully may represent effective therapeutic strategies for treatment of autoimmune and autoinflammatory disorders.

## Author contributions

Conceptualization, PL and VR; data curation, PL; writing, PL, supervision, VR. All authors contributed to the article and approved the submitted version.

## Conflict of interest

The authors declare that the research was conducted in the absence of any commercial or financial relationships that could be construed as a potential conflict of interest.

## Publisher’s note

All claims expressed in this article are solely those of the authors and do not necessarily represent those of their affiliated organizations, or those of the publisher, the editors and the reviewers. Any product that may be evaluated in this article, or claim that may be made by its manufacturer, is not guaranteed or endorsed by the publisher.
